# Morbidity and oncological outcomes after intersphincteric resection of the rectum for low-lying rectal cancer: experience of a single center in a lower-middle-income country

**DOI:** 10.1186/s12893-023-01940-9

**Published:** 2023-02-19

**Authors:** Antoinette Afua Asiedua Bediako-Bowan, Narious Naalane, Jonathan C. B. Dakubo

**Affiliations:** 1 Department of Surgery, University of Ghana Medical School, University of Ghana, P. O. Box 4236, Accra, Ghana; 2 Department of Surgery, Korle Bu Teaching Hospital, Accra, Ghana; 3 Mwin Tuba Hospital and Coloproctology Centre, Accra, Ghana

**Keywords:** Rectal cancer, Intersphincteric resection of rectum, Surgery, Local recurrence, Complications

## Abstract

**Background:**

Intersphincteric resection (ISR) of the rectum for low-lying rectal cancer with colo-anal anastomosis was introduced years ago, allowing for bowel continuity, and avoiding permanent stomas. The colorectal unit of Korle Bu Teaching Hospital adopted this procedure in 2014 when indicated, for the management of rectal cancers, where hitherto, abdominoperineal resection of the rectum with a permanent stoma was indicated. This study aimed to assess morbidity, mortality, and oncological outcomes associated with ISR of the rectum and determine the factors contributing to these.

**Methods:**

This was an observational study from prospectively stored data. All patients who underwent intersphincteric resection of the rectum due to low-lying rectal cancer from July 2014 to June 2021 were included in the study, and their records were assessed for intra-operative and 30-day postoperative complications, as well as mortality and their related risk factors and their oncological outcomes in terms of local recurrence at one year.

**Results:**

102 patients were included in this analysis. Six percent (6/102) of patients had intra-operative complications, including bleeding, and 41% (42/102) had 30-day postoperative complications, which were associated with pelvic side wall attachment of tumor and intra-op complications. Mortality risk was 12.7% (13/102) in the early postoperative period, and nine patients had a local recurrence within the first year of surgery.

**Conclusion:**

There is a high risk of early postoperative morbidity and mortality after intersphincteric resection of the rectum in our setting. The oncological outcomes are favorable in a population that abhors a permanent colostomy.

## Background

Cancers of the large bowel are a significant cause of morbidity and mortality, being the third most typical cancer diagnosed and the second cause of cancer-related deaths worldwide, with an estimated 1.9 million new cases and 915,880 deaths [1–4]. The incidence distribution of CRC in the bowel is; a third each for the right colon, left colon, and rectum [5]. In developing countries, close to 50% of colorectal cancers are located in the rectum, and of these, about 80% of them are low-lying and thus palpable on Digital Rectal Examination [6–9].

Surgery is critical in treating rectal cancers and has appreciable cure rates as a sole treatment, though alone, it is inferior to surgery with adjunct therapies. The surgeries for rectal cancer have evolved over the years; from being perineal to becoming abdominal and to a combination of the two; from being a mutilating pelvic surgery (causing impotence in all male patients) [10] to less mutilating forms; and from being solely open surgery to laparoscopic or robotic in recent times [11]. Understanding the anatomic role of the mesorectum in rectal cancer spread and the introduction of Total Mesorectal Excision (TME) in oncological surgery for middle and low-lying rectal cancer improved oncological outcomes while helping to preserve the anal sphincter [12].

Cancers of the rectum spread within the bowel wall in the longitudinal axis, circumferentially and radially through the muscularis propria to the serosa or adventitia. The limitation in the longitudinal spread has greatly influenced anal sphincter conservation in rectal cancer surgery. William et al. [13] and Pollet and Nicholls [14] proposed that perhaps a 2 cm distal resection margin was justifiable. To avoid a permanent colostomy, Parks introduced the low anterior resection of the rectum with a hand-sewn colo-anal anastomosis in 1972 [15], which was further improved with the introduction of staplers into gastrointestinal surgery.

Over the years, strides have been made enabling radical resection of the rectum and mesorectum with preservation of the anal sphincter, allowing for bowel continuity and avoiding permanent stomas. Schiessel and his colleagues demonstrated in 1994 that oncological resection for low-lying rectal cancer (between 1 and 5 cm from the anal verge) was feasible using the intersphincteric approach [11, 16]. In 2007, laparoscopic transanal rectal resection with coloanal anastomosis was also described [17].

This study aimed to assess early morbidity and mortality associated with and oncological outcomes regarding local recurrence following intersphincteric resection for low-lying rectal cancers and determine the factors contributing to these.

## Methods

This was a retrospective observational single cohort study from prospectively stored data by the Colorectal Surgery Unit of the Korle Bu Teaching Hospital. The colorectal unit of Korle Bu Teaching Hospital adopted the intersphincteric resection of the rectum for low-lying rectal cancer in 2014, when indicated, for the management of rectal cancers, where hitherto, abdominoperineal resection of the rectum with a permanent stoma was indicated. Korle Bu Teaching hospital is a 2000-capacity tertiary hospital in Ghana and serves the southern sector of the country as well as neighboring countries.

The study included patients diagnosed with rectal cancer by colonoscopy and histological confirmation and who had an intersphincteric resection of the rectum with immediate coloanal anastomosis from July 2014 to June 2021 (seven years). All patients had an abdominopelvic magnetic resonance imaging (MRI) or computerized tomography (CT) scan done to assess the depth of local spread, the liver, lymphadenopathy, and the involvement of other abdominal organs, as well as a chest x-ray or CT scan of the chest for the staging of the disease.

Patients had a short course (25 Gy in five fractions over 5 days) or long course (50.4 Gy in 28 fractions over 5 weeks, concurrently with capecitabine) neoadjuvant chemoradiation as prescribed by the board-certified radiation oncologists, based on the national comprehensive cancer network (NCCN) guidelines. All patients were given neoadjuvant chemoradiation unless a discussion at a weekly multidisciplinary team (MDT) meeting suggested otherwise. Few patients with T1 lesions, per MRI images, did not have neoadjuvant chemoradiation. Criteria for offering ISR in the unit include low rectal cancer in the distal 3rd of the rectum, which hitherto would have had an abdominoperineal resection. Contraindications to an ISR were patients who had a loss of anal sphincter function ab initio and those who had infiltration of anal sphincters per MRI imaging.

**Fig. 1:**
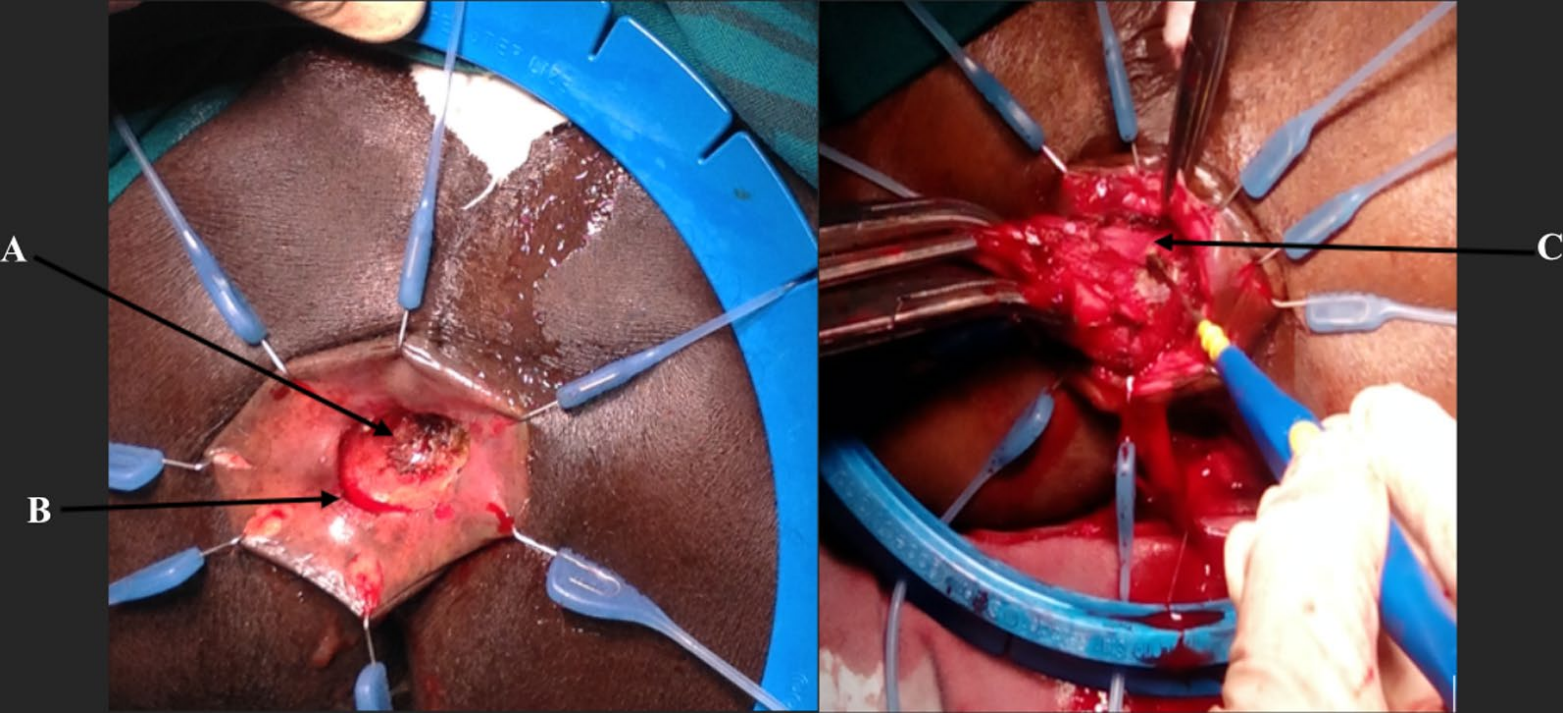
The transanal approach of intersphincteric resection of the rectum. The picture shows the anus retracted with a rectal tumor (arrowed **A**). **A** circumferential incision distal to the rectal tumor (arrowed **B**) and deepened into the intersphincteric plane to begin the dissection. The anus and rectum are dissected proximally along the intersphincteric plane (arrowed **C**) until the abdominal dissection plane is met within the pelvis

All surgeries were open procedures. With patients in the Lloyd-Davies position, it involved a synchronous combined open abdominal total mesorectal excision of the proximal rectum up to the level of the pelvic floor and a transanal dissection of the anus and distal rectum via the intersphincteric plane to meet the abdominal surgeon and remove the rectum/anus in one piece (Fig. 1). A few patients with rectal tumors in the middle third had an ISR due to the difficulty in performing an anastomosis within the abdomen after resection. A partial ISR was done unless tumor was within a centimeter of the anal dentate line, where a total ISR was performed. None of the patients had a defunctioning ileostomy.

Board-certified pathologists assessed the pathological specimen using the AJCC 8th edition guidelines, and based on the pathological staging, adjuvant chemotherapy regimens, comprising 6 to 12 cycles of a combination of capecitabine, folinic acid, oxaliplatin, and irinotecan were prescribed by the radiation oncologists based on NCCN guidelines.

This study assessed early postoperative complications, 30-day and overall mortality, risks for morbidity and mortality, and the one-year oncological outcomes in terms of local recurrence.

For this study, we included all patients who have undergone intersphincteric resection of the rectum due to low-lying rectal cancer from 1st July 2014 to 30th June 2021. We excluded patients who underwent intersphincteric resection of the rectum for benign causes or underwent other surgical procedures for rectal cancer. Each patient was followed up for at least one year.

Relevant information retrieved for this study included patients’ age, gender, presence of comorbidities, tumor location, histology type of the tumor, clinical stage of the disease, neoadjuvant therapy, presence of metastasis, date of surgery, intra-operative outcomes, early (30-day) postoperative complications, 30-day mortality, and local recurrence at one year. The data for this study was extracted from the register using a pre-designed datasheet and analyzed. No patient identifiers were used, and data were coded to protect the confidentiality of patients.

Data were validated and analyzed using Stata/MP version 16.1 ((Stata Corp., College Station, TX, USA). Logistics regression using the binomial model was used to test for the association of intra-operative morbidity, early post-op morbidity, and mortality, and local recurrence and risk factors of interest. A Kaplan–Meier estimation of local recurrence over the study period was done, taking into consideration 30-day mortality. A p-value ≤ 0.05 was considered statistically significant.

## Results

**Fig. 2:**
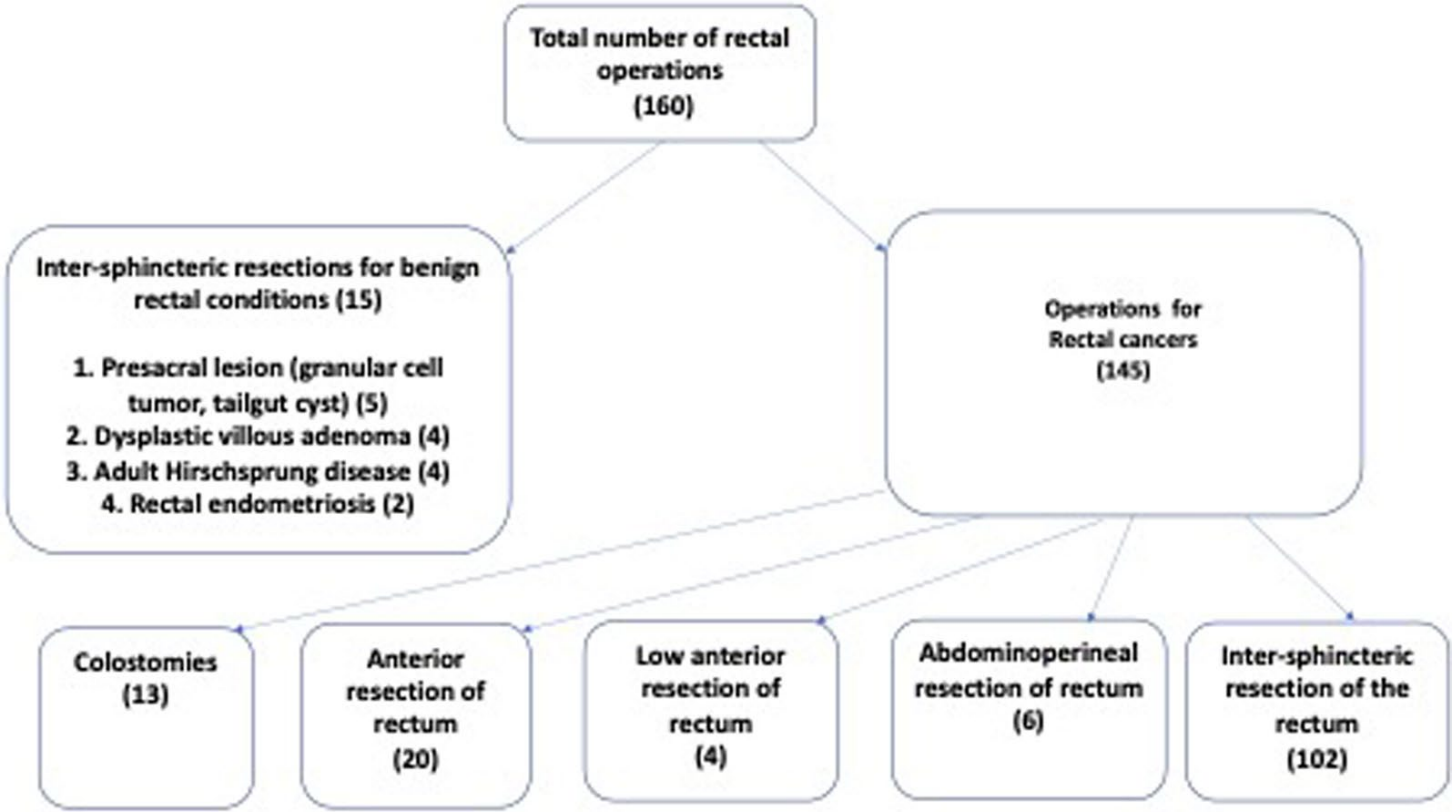
Flowchart of rectal cancer surgeries performed. The types of surgeries performed for rectal lesions in the colorectal unit

Over the study period, 160 patients had surgery for rectal lesions, 145 due to rectal cancer, of which 102 had intersphincteric resection of the rectum (ISR) (Fig. 2) were included in this analysis. Fifteen patients had intersphincteric resection of the rectum for benign conditions and were excluded from the analysis. Of the 102 patients who had ISR, 6% (6/102) had intra-operative complications, 41% (42/102) had early postoperative complications, and 9 had a local recurrence within one year of surgery.

The median follow-up period was 29 months (Interquartile range (IQR): 12–49 months), ranging between 0 to 92 months.

### Demographics/Characteristics

There was a 1:1 male: female ratio of patients, with a median age of 51 years (range 17–79 years; IQR: 40-61 years). A third of patients (36/102, 35%) were in the youthful age group, a quarter was elderly (26/102, 25%), and the rest were middle-aged 45-60 years (40/102, 39%) (Table 1). Twenty-eight percent (28/102) of patients had comorbidities, including hypertension (17/28), diabetes (1/28), chronic renal disease (2/28), and ulcerative colitis (1/28).

**Table 1:** Characteristics of patients with rectal cancers undergoing intersphincteric resection of the rectum

Characteristics	Number (%) (N = 102)
Gender	
Female	52 (50.98)
Male	50 (49.02)
Age	
< 45 years	36 (35.29)
45–60 years	40 (39.22)
> 60 years	26 (25.49)
Comorbidities	
No	74 (72.55)
Yes	28 (27.45)
Metastatic disease	
No	71 (69.61)
Yes	31 (30.39)
Neoadjuvant chemoradiation	
No	33 (32.35)
Yes, a short course	21 (20.59)
Yes, a long course	48 (47.06)
Clinical Stage of disease	
Stage 1	6 (5.88)
Stage 2	10 (9.80)
Stage 3	59 (57.84)
Stage 4	27 (26.47)
Adjuvant therapy	
Not applicable	13 (12.75)
No	27 (39.22)
Yes	62 (60.78)

The median distance of the tumor from the anal verge was 5 cm (Range 0.5 cm—10 cm, IQR 2–8 cm). About 70% (69/102) of participants had neoadjuvant chemoradiation therapy before rectal surgery. The most frequent histological diagnosis was adenocarcinoma (96%, 98/102), the others included malignant leiomyosarcoma, granular cell carcinoma, gastrointestinal stromal tumor, and a neuroendocrine tumor. Sixteen percent (16/102) of patients presented with stage 1 and stage 2 rectal cancer, and 84% (86/102) had stage 3 and 4 rectal cancers.

A third (31/102) of patients had metastatic disease (to the liver, lung, bone, and peritoneal seedlings) at presentation, and surgery was not aimed at a cure (Table 1). Surgery for the rest (71/102) of the patients aimed at curing. Seventy percent (62/89) of eligible patients had adjuvant chemotherapy after surgery.

**Fig. 3:**
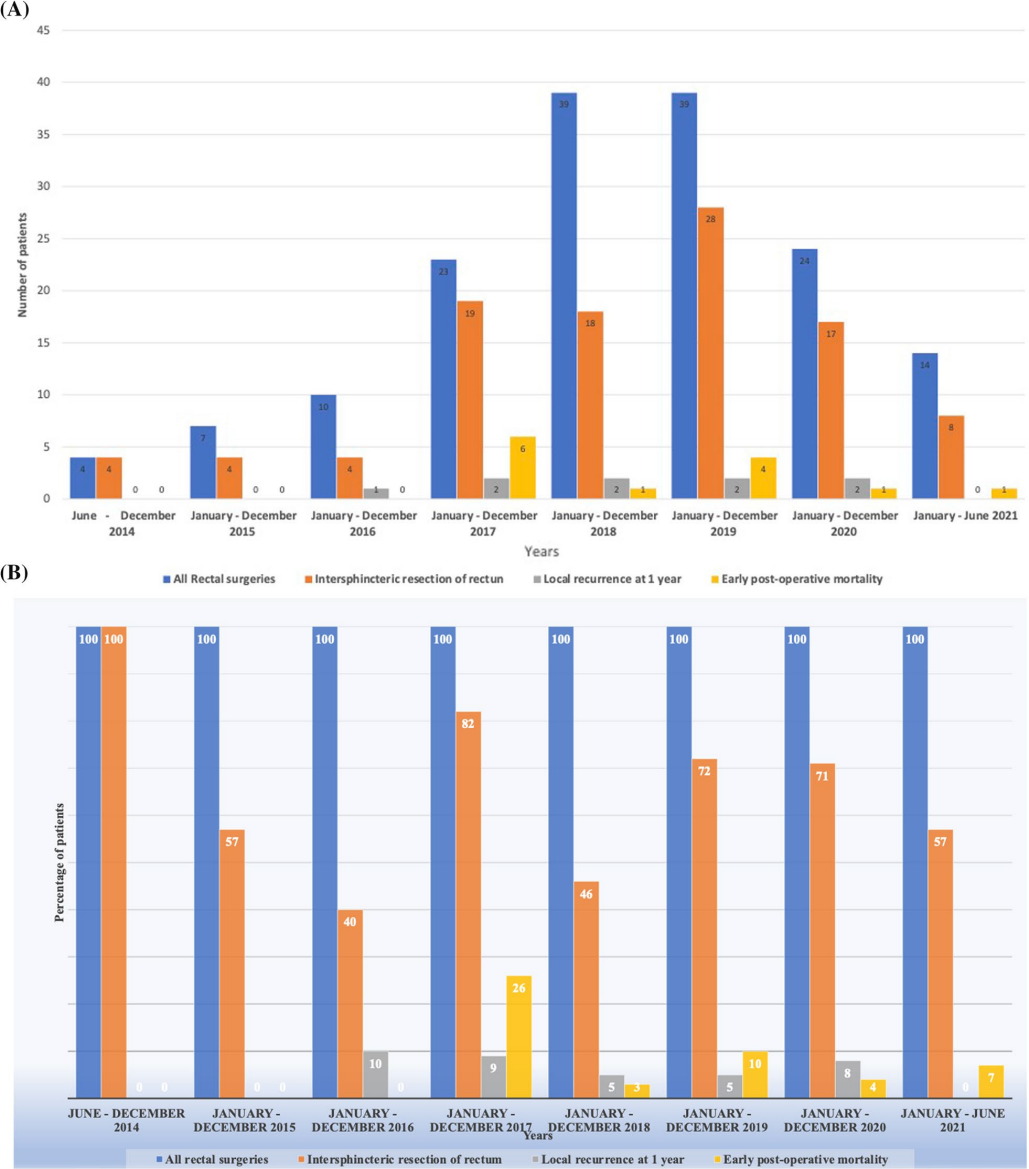
Trends in the number of intersphincteric resections of rectum over the years. **A** The number of intersphincteric resection of rectum of all rectal surgeries performed per year over the study period. Blue bars indicate the total rectal surgeries performed, orange bars indicate the number of intersphincteric resections of the rectum performed, the gray bars indicate those with local recurrence over the years, and yellow bars show those who had morbidity within 30 days after surgery. **B** The proportion of intersphincteric resection of rectum of all rectal surgeries performed per year over the study period. Blue bars indicate the total rectal surgeries performed, orange bars indicate the percentage of intersphincteric resections of the rectum performed, the gray bars indicate those with local recurrence over the years, and yellow bars show those who had morbidity within 30 days after surgery

Two of the patients had total ISR and the rest had partial ISR. The number of patients who underwent rectal surgeries increased from 2014 until 2021, when the numbers reduced due to the Covid-19 pandemic, and hospital admissions and elective surgeries were reduced (Fig. 3A).

### Morbidity

Six percent (6/102) of patients had intra-operative morbidity, the commonest being primary hemorrhage. Three of these died within the 30-day period, and two were managed in the intensive care unit (ICU). There was a seven-fold risk of intra-operative morbidity (bleeding), when the tumor was attached to the pelvic side wall (OR: 7.142 (CI: 1.222–41.725), p = 0.029). The risk of intra-operative morbidity, though higher, was not significantly related to increasing age (p = 0.464), the presence of comorbidity (p = 0.219), a higher stage of the disease (p = 0.354) or having neoadjuvant therapy before surgery (p = 0.412) (Table 2).

**Table 2:** Tumor characteristics associated with intra-operative morbidity

Characteristics (Number of patients; N = 102)	Number of patients with intra-operative morbidity (%)	Odds ratio (Confidence interval)	p-value
Age			0.464
< 50 years (49)	2 (4.1)		
> 50 years (53)	4 (7.5)	1.918 (0.335–10.995)	
Comorbidity			0.219
No (74)	3 (4.1)		
Yes (28)	3 (10.7)	2.841 (0.537–14.995)	
Pelvic attachment			0.029
No (77)	2 (2.6)		
Yes (25)	4 (16.0)	7.142 (1.222–41.725)	
Stage of disease			0.354
Stage 1 & 2 (16)	0		
Stage 3 & 4 (86)	6 (7.0)	1.826 (0.511–6.523)	
Neoadjuvant Chemoradiation			0.412
No (33)	1 (3.0)		
Yes (69)	5 (7.2)	2.500 (0.280–22.306)	
Surgical Procedure			-
ISR only (92)	6 (6.5)		
ISR + extended organ resection (10)	0	–	

*ISR* Inter-sphincteric resection of the rectum

Forty-two (42/102) percent of patients had an increased risk of morbidity within 30 days of surgery. Postoperative morbidity included surgical site infections, pneumonia, cardiac events, necrosis of the neorectum, and the intestinal pouch created. Eleven were managed conservatively or with procedures under local anesthesia (Clavien Dindo (CD) I, II & IIIA), twenty had reoperation under general anesthesia (CD IIIB), two needed ICU (CD IV) care and nine died (CD V). Those who underwent an ISR with an extended organ resection had a seven-fold risk of developing early postoperative morbidity (OR: 6.823 (CI:1.369–34.009), p = 0.019) (Table 3). Partial urinary bladder excision, hysterectomy, and anterior vaginectomy were the commonly extended organ resections that were done. However, the presence of metastasis to any site was not significantly associated with postoperative morbidity, neither was the stage of rectal cancer (p = 0.613) nor having had neoadjuvant chemoradiation (p = 0.340) (Table 3).

**Table 3:** Tumor characteristics associated with 30-day postoperative morbidity

Characteristics (Number of patients; N = 102)	Number of patients with intra-operative morbidity (%)	Odd’s ratio	p-value
Liver metastasis			0.207
No (90)	35 (38.9)	–	
Yes (12)	7 (58.3)	2.200 (0.647–7.477)	
Lung metastasis			0.716
No (98)	40 (40.8)	–	
Yes (4)	2 (50.0)	1.450 (0.196–10.724)	
Bone metastasis			0.060
No (101)	41 (40.6)	–	
Yes (1)	1 (100.0)	1.000 (0.459–1.016)	
Peritoneal seedlings			0.215
No (94)	37 (39.4)	–	
Yes (8)	5 (62.5)	2.567 (0.578–11.392)	
Ascites present			0.925
No (95)	39 (41.1)		
Yes (7)	3 (42.9)	1.076 (0.228–5.082)	
Pelvic attachment			0.208
No (77)	29 (37.7)	–	
Yes (25)	13 (52.0)	1.793 (0.721–4.454)	
Stage of disease			0.613
Stage 1 (6)	4 (66.7)	–	
Stage 2 (10)	4 (40.0)	0.333 (0.040–2.768)	
Stage 3 (59)	18 (30.5)	0.219 (0.036–1.308)	
Stage 4 (27)	16 (59.3)	0.727 (0.112–4.685)	
Neoadjuvant chemoradiation			0.340
No (33)	16 (48.5)	–	
Yes, a short course (21)	8 (38.1)	0.653 (0.214–1.992)	
Yes, a long course (48)	18 (37.5)	0.637 (0.259–1.565)	
Surgical procedure			0.019**
ISR only (92)	34 (36.9)	–	
ISR + extended organ resection (10)	8 (80.0)	6.823 (1.369–34.009)	

*ISR* Inter-sphincteric resection of the rectum. **Characteristics with significant association with 30-day postoperative morbidity

### Mortality

There was a 12.75% (13/102) risk of early (30-day) postoperative mortality, and this was significantly related to patients who developed intra-operative complications (OR 8.6; CI:1.526–48.465; p = 0.015) and postoperative complications (OR 5.937; CI:1.522–23.154; p = 0.010).

The overall mortality for the follow-up period was 36.27% (37/102). The range of months to death was 0–48 months, with a median duration of 6 months after surgery. Mortality was due to intra- or postoperative morbidity in 13 of the 37 patients, and to tumor progression in the rest (24/37).

### Local tumor recurrence

Nine patients developed local recurrence within one year of ISSR. The percentage of patients with local recurrence was on average 5 percent of patients who had rectal surgery reduced over the years (Fig. 3B).

**Fig. 4:**
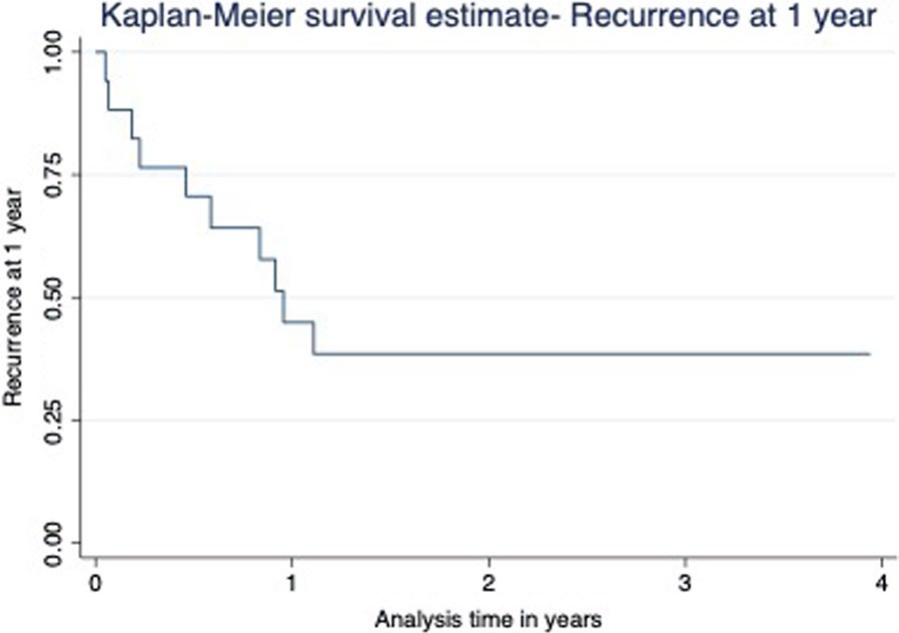
Kaplan–Meier survival estimate for recurrence of low-lying rectal cancers. Kaplan–Meier survival estimate in terms of tumor recurrence at one year taking into consideration, mortality over the years

Local recurrence was significantly related to the attachment of the tumor to the pelvic sidewall at surgery (OR 4.562; CI:1.119–18.590; P = 0.034). Factoring in mortality, recurrence at one year was 40% (Fig. 4).

At two years post-surgery, six persons also reported a local recurrence. No recurrence was recorded for years three to seven, post-surgery. At the time of analysis, of the four persons who had ISR seven years prior in 2014, one died of metastatic disease (had lung metastasis at surgery) within one year of surgery, and the rest are alive with no local or distant tumor recurrence: one had a re-operation within 30 days of surgery on account of pouch necrosis at the neo-rectoanal anastomosis and given a permanent colostomy.

## Discussion

Over the study period, 102 patients with rectal cancer were studied who had intersphincteric resection of the rectum with bowel continuity preserved, 41% had 30-day postoperative morbidity, 6% intra-operatively, there was a 12% 30-day mortality risk, and nine patients had a local recurrence of the tumor within one year.

This is an initial report from a newly established colorectal surgery unit in a teaching hospital where intersphincteric resection of the rectum with primary coloanal anastomosis was adopted to encourage patients to accept surgical treatment for low rectal cancer.

This study showed a male: female ratio of 1:1, similar to the 1.3:1 global trend [4, 18, 19]. This study's patients were a younger cohort with a median age of 51 years, whereas globally, patients are usually in their sixth decade of life at presentation [4, 19].

A more significant proportion of patients (80%) in this study presented with stage 3 and stage 4 disease, a third of them with stage 4 disease. There are no national screening programs for colorectal cancers in the country, and patients present late for treatment. Patients with low rectal cancers were offered abdominoperineal resection of the rectum with a permanent colostomy, which further made them default treatment, presenting later with disease progression. Hence the move to providing patients with low rectal cancers sphincter-sparing procedures with bowel continuity in our setting. A third of the patients in this study had metastatic disease (to the liver, lung, bone, and peritoneal seedlings or with a tumor attached to the pelvic side wall) at presentation.

This study reports a low number of patients with rectal cancers and rectal cancer surgeries in the initial years, with the numbers increasing as the years progressed. This is because patients with a rectal tumor in our hospital tended to refuse surgery when offered abdominoperineal resection for low-lying tumors and defaulted treatment. Schiessel and his colleagues demonstrated in 1994 that oncological resection for low-lying rectal cancer (between 5 and 1 cm from the anal verge) was feasible using the Intersphincteric approach [16]. The principles underpinning success in rectal cancer surgery are the removal of the primary tumor with adequate circumferential margin and the regional lymph nodes and reconstruction /restoration of intestinal continuity with or without sphincter preservation [20, 21]. The introduction of intersphincteric resection of the rectum for low-lying rectal tumors and bowel continuity in our hospital has, over the years, improved the number of patients who accept the management of low rectal cancers.

The introduction of the neoadjuvant radiotherapy [22] and neoadjuvant chemoradiation [23] in the early 2000s, along with total mesorectal excision for rectal tumors with optimal circumferential resection margins, have helped in the control of recurrence post-surgical resection [12, 21]. Nearly 70% of the patients in this study had neoadjuvant chemoradiotherapy. Neoadjuvant chemoradiotherapy has been associated with increased risk of anastomotic leaks after surgery and are not advocated for in early-stage rectal tumors [24]. Neoadjuvant chemoradiation thus was not done for patients with early-stage 1 or 2 tumors after discussion at an MDT meeting for colorectal cancers or for those who presented to the surgical unit with a complication requiring immediate surgery.

Intra-operative morbidity occurred in 6% and 30-day morbidity in 41% of the participants. Intersphincteric resection of the rectum was introduced to our unit in 2014 and performed by two general surgeons interested in colorectal diseases over the period. The team has seen improvement in surgical technique over the years, with an increase in the number of procedures and improvement in clinical outcomes. There have been significant strides in intersphincteric resection of the rectum in Europe, Japan, and some Asian countries since 2000. After this procedure, many reports have been made on the clinical, operative, oncological, and quality of life. Treatment outcomes are best in high-volume centers with expertise in colorectal cancer care. This notwithstanding, significant morbidity after surgery still occurs. A wide prevalence rate of 7.5–38.3% [25] has been reported, reflecting the varied availability of expertise and resources in treating rectal cancer in different settings.

Defunctioning stomas were not fashioned out for patients in this study. Patients refused to have a stoma and on the other hand, anastomotic leaks could be accessed per the anal canal. Defunctioning stomas, though they cannot prevent an anastomotic leak, traditionally is fashioned out after ISR to protect the anastomotic site in 100 percent of cases [25].

Significant intra-operative injuries/complications observed in rectal cancer surgery include hemorrhage, ureteric, urinary bladder, and bowel injuries. Post-operative complications after rectal cancer surgery, which impact heavily on treatment outcomes, include sepsis from surgical site infections (Deep and organ space), deep vein thrombosis/pulmonary embolism, and cardiac and respiratory failures. Fistulae between the bowel, urinary bladder, and vagina are also common. Intra-operative complications in our study cohort included primary hemorrhage and urinary bladder injury; postoperative complications included surgical site infections, pneumonia, cardiac events, and necrosis of the neorectum, and the intestinal pouches created.

A third of mortality over the study period occurred in the 30 days. It was associated with intra-operative and post-operative morbidity, and two-thirds of mortality recorded was due to tumor progression. This may be explained by the fact that most patients presented late with stage 3 and stage 4 disease and some were metastatic. In low- and middle-income countries, patients tend to report late symptoms. Patients tend to overlook signs that may suggest a malignancy of the rectum, such as bleeding per rectum, attributing it to hemorrhoids, and seeking herbal treatment until it is late.

Nine patients had a local recurrence within one year of surgery. The mortalities within the year were factored in analyzing the recurrence rate, hence fewer persons were the denominator for calculating the recurrence rate at 1 year. The high recurrence rate may also be explained by the fact that more than 80% of patients were either stage III or stage IV disease, with tumor progression. Though this may be higher than in high-volume centers, it is encouraging for patients with low-lying rectal cancers in our setting. Local recurrence was seen within two years of surgery, after which participants were noticed to have stable disease over the rest of the seven-year study period. The association of local recurrence with pelvic wall attachment at the surgery in our cohort suggests a need to be selective in offering patients ISR. This is similar to recent studies indicating a high incidence of local recurrence due to lateral spread rather than positive distal resection margin [14, 26, 27]. Despite the local recurrence, anal preservation was welcoming to our patients. A limitation of this study is that it does not describe the anal dysfunction that may have been experienced by patients after ISR. Studies suggest that, most importantly, patients do not want to have a permanent colostomy [28].

## Conclusion

There was a high risk of early post-operative morbidity associated with inter-sphincteric resection of the rectum with immediate coloanal anastomosis. The oncological outcomes are relatively favorable, though, and there is a need to reassess the criteria for selection for this procedure and a need for intensified training for this procedure.

## Data Availability

Data is available on request to the corresponding author.

## Abbreviations

CI: Confidence interval
IQR: Interquartile range
ISR: Intersphincteric resection of the rectum
KBTH: Korle Bu Teaching Hospital
LMIC: Low- and middle-income country
